# MicroRNA-5195-3p mediated malignant biological behaviour of insulin-resistant liver cancer cells via SOX9 and TPM4

**DOI:** 10.1186/s12885-023-11068-x

**Published:** 2023-06-16

**Authors:** Jing Yan, Bei Xie, Ye Tian, Wenqin An, Zhiheng Peng, Zhuan Liu, Jing Li, Linjing Li

**Affiliations:** 1 Department of Clinical Laboratory Center, The Second Hospital of Lanzhou University, Lanzhou, 730000 Gansu China; 2 Department of Clinical Laboratory Center, Gansu Provincial Maternity and Child-care Hospital (Gansu Province Central Hospital), Lanzhou, 730000 Gansu China; 3 Department of Medical Laboratory Animal Science, School of Basic Medical Sciences, Lanzhou University, Lanzhou, 730000 Gansu China; 4 The First School of Clinical Medicine, Lanzhou University, Lanzhou, 730000 Gansu China

**Keywords:** Liver cancer, Insulin resistance, miR-5195-3p, Malignant biological behaviour, SOX9, TPM4

## Abstract

**Background:**

Primary liver cancer is a malignant tumour of the digestive system, ranking second in cancer mortality in China. In different types of cancer, such as liver cancer, microRNAs (miRNAs) have been shown to be dysregulated. However, little is known about the role of miR-5195-3p in insulin-resistant liver cancer.

**Methods and results:**

In this study, in vitro and in vivo experiments were conducted to identify the altered biological behaviour of insulin-resistant hepatoma cells (HepG2/IR), and we proved that HepG2/IR cells had stronger malignant biological behaviour. Functional experiments showed that enhanced expression of miR-5195-3p could inhibit the proliferation, migration, invasion, epithelial-mesenchymal transition (EMT) and chemoresistance of HepG2/IR cells, while impaired expression of miR-5195-3p in HepG2 cells resulted in the opposite effects. Bioinformatics prediction and dual luciferase reporter gene assays proved that SOX9 and TPM4 were the target genes of miR-5195-3p in hepatoma cells.

**Conclusions:**

In conclusion, our study demonstrated that miR-5195-3p plays a critical role in insulin-resistant hepatoma cells and might be a potential therapeutic target for liver cancer.

**Supplementary Information:**

The online version contains supplementary material available at 10.1186/s12885-023-11068-x.

## Introduction

Primary liver cancer, ranking as the second leading cause of cancer mortality in China, is a common malignant tumour of the digestive system [1, 2]. Normally, the majority of liver cancer patients are diagnosed at an advanced stage, which contributes to a higher rate of postoperative relapse and metastasis [3]. Insulin resistance (IR), a chronic pathological process, is characterized by decreased glucose uptake and utilization and excessive insulin secretion in individuals [4]. Numerous studies have shown that high levels of insulin, especially in patients with IR, are significantly associated with an increased risk of various tumours, including liver cancer [5–7]. A variety of pathological conditions, such as liver cancer, lead to dysfunction of the insulin signalling pathway in liver cells, which reduces their sensitivity to insulin and leads to IR [8]. However, IR can increase the level of hepatic glucose to form a vicious cycle, aggravating the progression of IR [9]. In previous studies, we employed a high concentration of insulin to induce HepG2 cells to establish a stable insulin-resistant cell model (HepG2/IR) and explored the relationship between IR and chemoresistance in liver cancer cells [10]. However, few studies have focused on the relationship and mechanism between IR and the malignant biological behaviour of liver cancer cells.

microRNAs (miRNAs) are highly conserved short-chain endogenous noncoding RNAs that bind to the 3′-UTR (untranslated region) of target mRNAs and regulate gene expression [11]. A majority of studies have shown that miRNAs are associated with the occurrence and development of tumours [12, 13]. Growing evidence has also shown that miRNAs play an important role in liver cancer. miR-21, for example, was found to be upregulated in hepatocellular carcinoma (HCC), and interference of miR-21 inhibited its proliferation [14]. However, the mechanism of miRNAs in insulin-resistant hepatocarcinoma remains unclear. Our previous study showed that miR-5195-3p was differentially downregulated in HepG2/IR cells using miRNA expression profiling [15]. Only a few studies on miR-5195-3p have been reported, which indicated that it could inhibit cell proliferation, metastasis and invasion in non-small cell lung cancer and triple-negative breast cancer [16, 17]. However, the relationship between miR-5195-3p and insulin-resistant liver cancer cells remains unclear. There is an urgent need to explore the underlying regulatory mechanisms.

In the present study, we found that elevated expression of miR-5195-3p could inhibit growth, migration, invasion, drug resistance, and epithelial-mesenchymal transition (EMT) in vitro and in vivo in HepG2/IR cells, in contrast to its downregulation. Further studies showed that miR-5195-3p negatively regulated malignant biological behaviour in HCC by targeting sex determining region Y-box 9 (SOX9), which could interact with a variety of downstream proteins and exhibit stimulatory or inhibitory activity in different types of cancer cells [18], and tropomyosin 4 (TPM4), which is abnormally expressed in a variety of cancers [19]. Thus, this study provides new potential biomarkers and therapeutic targets in liver cancer.

## Materials and methods

### Cell culture and induction to IR

HepG2 and 293T cells were purchased from the American Tissue Culture Collection (ATCC) and cultured with Dulbecco’s modified Eagle’s medium (DMEM, HyClone, USA) with 10% foetal bovine serum (FBS, HyClone, USA) at 37 °C in a 5% CO_2_ incubator. The induction process of insulin-resistant cells was as follows: cells were synchronized in serum-free DMEM for 6 h (h) after they had completely adhered. DMEM containing 5% calf serum was replaced, and 0.2 µmol/L insulin (Sigma, USA) was added to induce 72 h. The induced insulin-resistant cells were named HepG2/IR cells [10].

### Cell transfection

For the miR-5195-3p functional analysis, miR-5195-3p mimic and NC mimic (Ruibo, China) were transfected into HepG2/IR cells using Lipofectamine 2000 (Invitrogen, USA) according to the manufacturer’s protocols. In the same way, HepG2 cells were transfected with miR-5195-3p inhibitor or NC inhibitor (Ruibo, China) for 48 h. Then, these cells were collected and subjected to further analysis.

### MTT (3-(4,5-dimethylthiazol-2-yl)-2,5-diphenyltetrazolium bromide) assay

Cells from different groups were plated in 96-well plates at a density of 1.2 × 10^4^ cells per well. The viability of the cells was determined by MTT assays 1, 2, 3, 4, 5 and 6 days (d) following seeding, followed by 4 h incubation with MTT solution. DMSO was added to each of the wells. The absorbance values of each well were measured at 490 nm, and the readings were quantified using a Powerwave X plate reader (Bio-Tek Instruments, USA).

The 50% inhibitory concentration (IC50) values were determined as the drug concentration causing 50% cell growth inhibition. The MTT assay was performed to assess cell sensitivity to mitomycin (MMC), oxaliplatin (OXA), vincristine (VCR) and sorafenib. Briefly, cells from different groups were seeded in 96-well plates at a density of 1.5 × 10^4^ cells per well and incubated with the above drugs for 48 and 72 h, and the cells were treated in a similar fashion as described above.

### Ethynyl-2’-deoxyuridine (EdU) proliferation assay

An EdU proliferation assay (Yeasen, China) was performed to measure cell proliferation. In brief, cells were plated in 96-well plates (2 × 10^3^ cells/well) with 100 µL of 10% serum-containing DMEM per well for 24 h. Then, the cells were cultured with 50 µM EdU in serum-free DMEM for 2 h at 37 °C, followed by fixation in 4% formaldehyde for 30 min on the second day. Glycine was used to neutralize formaldehyde. After permeabilization with 0.5% Triton X-100 for 10 min at room temperature, 1× Apollo reaction cocktail (100 µL) was added to the wells for 30 min. Nuclei were stained with 1× DAPI (100 µL). Finally, the cells were imaged under a fluorescence microscope (Olympus BX 60 fluorescence microscope, Japan).

### Scratch wound healing assay

Cell migration was determined using a scratch wound-healing assay. In brief, cells were cultured in 6-well plates to 80% confluence. Subsequently, the supernatant was discarded, and the cells in the 6-well plates were scratched with a 10 µL tip and incubated with serum-free DMEM for 24 h. Then, the cells were further cultured for 48 and 72 h. Cell migration was analysed by counting migrated cells under an inverted microscope (Olympus X51 Inverted Microscope, Japan) using ImageJ 1.8.0 (National Institutes of Health, USA).

### Colony formation unit assay

Cells were seeded in 6-well plates at a density of 1 × 10^3^ cells/well. DMEM was replaced every 4 d. After culture for 14 d, the cells were fixed with 4% paraformaldehyde for 15 min at room temperature and stained with crystal violet for 15 min at room temperature.

### Transwell assay

Cells (2 × 10^5^ cells/well) were added to the apical chamber, and 600 µL of 20% FBS-containing medium was added into the basolateral chamber. Next, the chambers were incubated at 37 °C for 48 h. The plate was rinsed and subsequently stained using 0.1% crystal violet at room temperature for 30 min. For the cell invasion assay, 40 µL of Matrigel (Shanghai YuBo Biological Technology, China) was added to the chamber of the transwell unit at 37 °C for 4.5 h to form a basement membrane, and cells were treated in a similar fashion as that in the cell migration assay.

### Flow cytometry assay

Apoptotic analysis was performed using an Annexin V/PI cell apoptosis kit (Invitrogen, USA). Samples were gently suspended in 100 µL of binding buffer containing 2.5 µL of Annexin V-FITC and 2.5 µL of propidium iodide (PI) and further incubated for 15 min in the dark at room temperature. Finally, cells were suspended in 500 µL of binding buffer and detected by flow cytometry using FACSVerse (BD Biosciences, USA). Flow cytometry data were analysed using FlowJo 10 (FlowJo). The apoptotic rate was determined for each condition as follows: apoptotic rate = (early apoptotic rate + late apoptotic rate) × 100%.

### Prediction of target genes of miR-5195-3p

miR-5195-3p target genes were analysed using *TargetScan* (http://www.targetscan.org/vert_80/). The functions of all these target genes were further screened by GenBank.

### RNA isolation and RT‒qPCR assay

Total RNA from the cells was isolated using TRIzol® reagent (Invitrogen, USA). For miRNA levels, detection and quantification of miRNAs from total RNA samples were performed using the Hairpin-it™ miRNA qPCR Quantitation Kit (Shanghai GenePharma, China) according to the manufacturer’s protocol. RT‒qPCR was performed using a Rotor-Gene 3000 quantitative PCR amplifier (Corbett Life Science, USA). The primers were purchased from Shanghai GenePharma (Shanghai, China): miR-5195-3p mimic, AUCCAGUUCUCUGAGGGGGCU, NC mimic, UUUGUACUACACAAAAGUACUG; and miR-5195-3p inhibitor, AACCCCUAAGGCAACUGGAUGG, NC inhibitor, CAGUACUUUUGUGUAGUACAAA. The miRNA concentration was normalized to the endogenous control U6. SOX9 (F: 5’-CGAGCTCGTATTCCTCACCCTAGATTTG-3’; R: 5’-CGACGCGTACAATATAAGGCAGCCCAA-3’) and TPM4 (F: 5’-CGAGCTCTCCATACTTCAGGGAACAGCAA-3’; R: 5’-CGACGCGTTAAGCCAGAAGCAGGGTG-3’) sequences were used.

For gene mRNA detection, total RNA was reverse transcribed to cDNA using a PrimeScript RT reagent kit purchased from TaKaRa Bio (Otsu, Japan) according to the manufacturer’s protocol. qPCR was performed using a SYBR Premix Ex Taq II kit (Toyobo, Japan). β-Actin was used as the internal control. The relative expression levels of the genes were determined by the 2^−ΔΔCt^ method.

### Western blot assay

After cells were lysed with RIPA lysis buffer (Beijing Solarbio Science & Technology, China), proteins were collected, and their concentrations were determined using the BCA method. The proteins (30 µg) were separated on 10% sodium dodecyl sulphate‒polyacrylamide gel electrophoresis (SDS‒PAGE) gels and were subsequently transferred onto polyvinylidene membranes (EMD Millipore, USA). Following blocking in 0.1% TBS-Tween 20 containing 5% nonfat milk at room temperature for 1 h, the membranes were incubated overnight at 4 °C with primary antibodies against SOX9 (1:2000; cat. no. ab185966; Abcam, UK), TPM4 (1:2000; cat. no. ab181085; Abcam, UK), E-cadherin (1:1000; cat. no. ab40772; Abcam, UK), N-cadherin (1:1000; cat. no. ab76011; Abcam, UK), Vimentin (1:1000; cat. no. ab92547; Abcam, UK), Snail (1:1000; cat. no. ab216347; Abcam, UK) and β-actin (1:1000; cat. no. TA-09; Zhongshan Jinqiao Bio-Technology, China). The following morning, the membranes were incubated at room temperature for 1 h with horseradish peroxidase-conjugated goat anti-mouse (1:5000; cat. no. SA00001-1; Proteintech Group, USA) or goat anti-rabbit (1:5000; cat. no. SA00001-2; Proteintech Group, USA) secondary antibodies and subsequently developed using an Amersham Enhanced Chemiluminescence Western blot detection system (GE Healthcare Life Sciences, USA) according to the manufacturer’s protocols.

### Dual luciferase reporter assay

Both the 3’-UTR of SOX9 and the 3’-UTR of TPM4, containing miR-5195-3p binding sites, were amplified, cloned and inserted into a pMIR-REPORT luciferase vector in sense or antisense directions using MluI and SacI at the restriction enzyme cutting sites. Then, 293T cells were seeded in 96-well plates 1 d before transfection. The 293T cells were cotransfected with SOX9 or TPM4 3’-UTR pMIR-REPORT luciferase vector, pRL-TK reporter vector and miR-5195-3p mimic or NC mimic using Lipofectamine 2000. Forty-eight hours after transfection, firefly and Renilla luciferase activities were measured using the Dual-Glo® Luciferase Assay System on a FlexStation 3 Multi-Mode Microplate Reader (Molecular Devices, USA) according to the manufacturer’s instructions. Relative luciferase activity was normalized to Renilla luciferase activity.

### In vivo xenograft mouse model

Male BALB/c nude mice (specific pathogen-free grade, 5 weeks old, 18–22 g) used in these experiments were purchased from Vital River Laboratories (Beijing, China). The experiments with animals were conducted in accordance with the Care and Use of Laboratory Animals published by the US National Institutes of Health. Mice were randomly divided into twelve groups (HepG2, NC inhibitor, miR-5195-3p inhibitor, HepG2 + OXA, NC inhibitor + OXA, miR-5195-3p inhibitor + OXA, HepG2/IR, NC mimic, miR-5195-3p mimic, HepG2/IR + OXA, NC mimic + OXA, miR-5195-3p mimic + OXA) and injected with transfected cells (2 × 10^6^ cells) through subcutaneous axillary injection and treated with 7 mg/kg OXA through intraperitoneal injection every week. After 30 d, the mice were euthanized with CO2, and the tumours were harvested, measured and weighed. The volume of the tumour was estimated by a ruler as follows: tumour volume = 0.5 × tumour length × tumour width^2^ [20].

### Statistical analyses

The data are presented as the mean ± standard deviation from at least triplicate experiments performed three times. Statistical analyses were performed using GraphPad Prism (version 6.07; GraphPad Prism Software, San Diego, CA). The difference between two groups was analysed using Student’s t test. Multiple comparisons were performed using ANOVA. P < 0.05 was considered to indicate a significant difference.

## Results

### HepG2/IR cells displayed enhanced proliferation, migration, invasion, EMT and chemoresistance

**Fig. 1:**
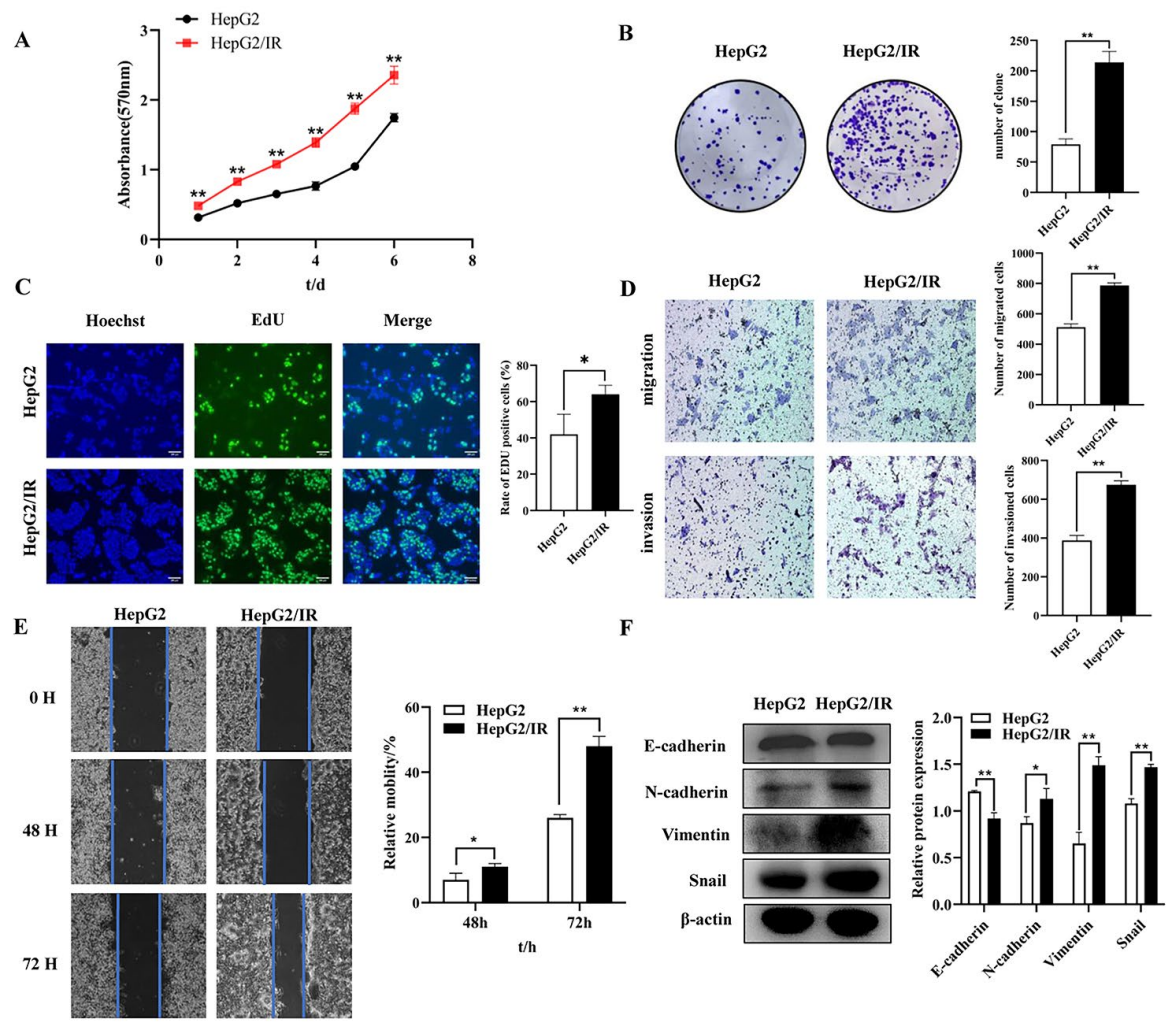
HepG2/IR cells displayed enhanced proliferation, migration, invasion and EMT. (**A**) MTT assays were performed with HepG2 and HepG2/IR cells. (**B**) Colony formation assays were performed with HepG2 and HepG2/IR cells. (**C**) DNA replication was detected by EdU assays in HepG2 and HepG2/IR cells. (**D**) Representative image of migration and invasion in HepG2 and HepG2/IR cells. (**E**) The migration of HepG2 and HepG2/IR cells was determined by scratch wound-healing assays. (**F**) The protein expression of E-cadherin, N-cadherin, Vimentin and Snail in HepG2 and HepG2/IR cells was determined by Western blotting; the membranes were cut prior to hybridization with antibodies. The experiments were independently repeated three times. (* P < 0.05, ** P < 0.01)

MTT, EdU and colony formation unit assays were used to detect the proliferation of HepG2/IR cells and their parental cells (control group). The MTT absorbance at 1–6 d indicated that the HepG2/IR cells showed enhanced proliferation compared with HepG2 cells (Fig. 1A). Moreover, colony formation assays showed that the proliferation increased in HepG2/IR cells (224.33 ± 12.66) compared with their parental cells (78.67 ± 8.74) (Fig. 1B). Additionally, an increased EdU-positive rate was observed in HepG2/IR cells (78.2 ± 11.12%) (Fig. 1C). To investigate the effects of IR on cell migration, invasion and EMT, we assessed HepG2 and HepG2/IR cells by wound-healing assays, transwell assays and Western blots. The transwell assay with or without Matrigel demonstrated that IR significantly induced the migration and invasion of HepG2/IR cells compared with those of the control group (Fig. 1D). The 48 and 72 h wound-healing assays revealed that the migratory capacity of HepG2/IR cells was increased significantly compared with that of the control group (Fig. 1E). As shown in Fig. 1F, compared with that of the control group, the protein expression of N-cadherin, Vimentin and Snail was significantly increased, while the protein E-cadherin was suppressed in HepG2/IR cells. Enhanced proliferation, migration, invasion and EMT ability in HepG2/IR cells indicated that IR could promote proliferation, migration, invasion and EMT in hepatoma cells.

**Fig. 2:**
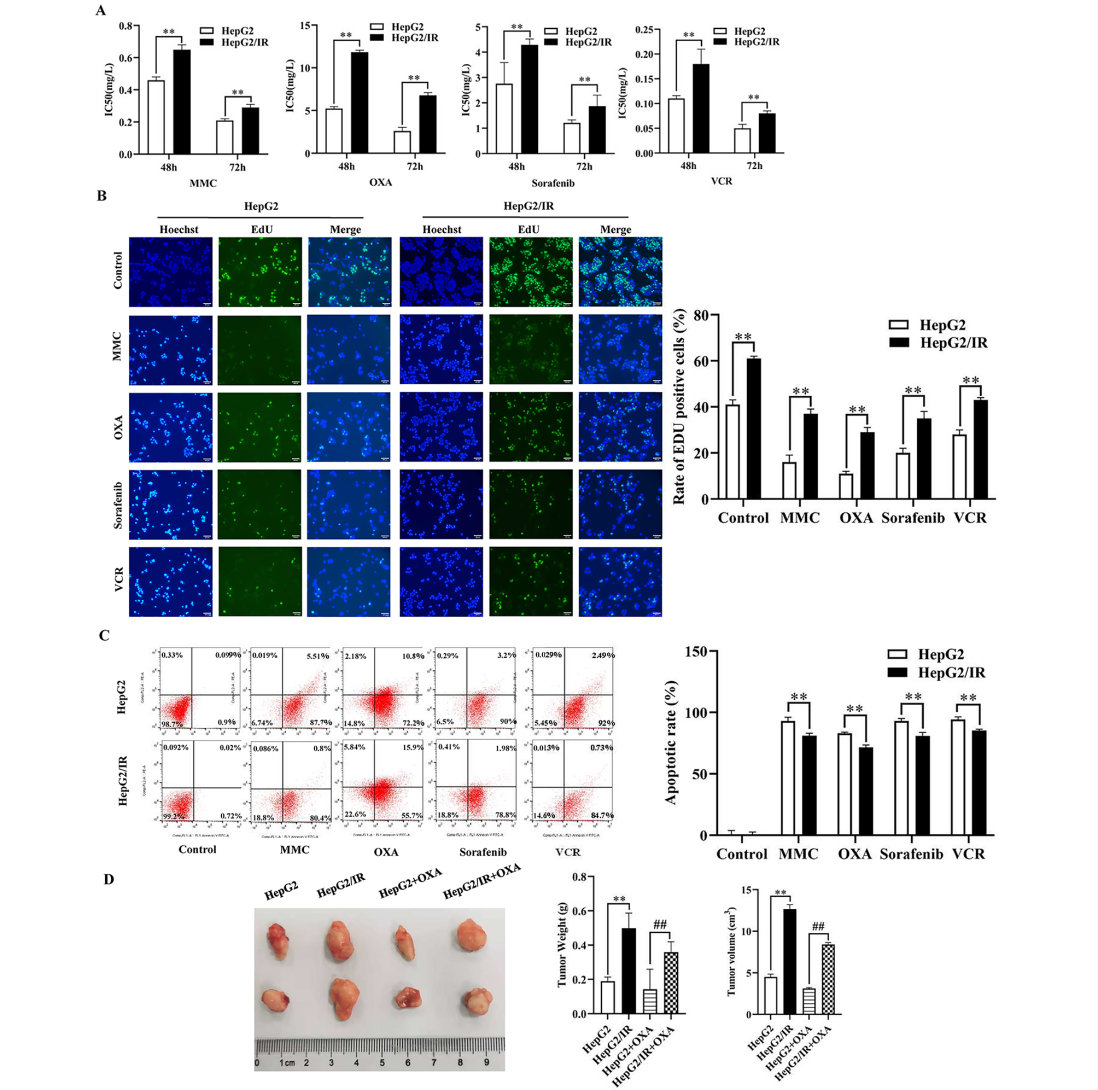
HepG2/IR cells displayed enhanced chemoresistance. The IC50 value (**A**) and EdU-positive rate (**B**) of MMC, VCR, OXA and sorafenib in HepG2/IR cells and their parental cells. (**C**). Representative images of apoptosis. D. The tumours dissected from all groups were photographed. (* P < 0.05, ** P < 0.01)

To investigate and illuminate the relationship between IR and chemoresistance in HepG2 cells, cell viability assays, EdU assays, apoptotic analyses, and in vivo and animal experiments were performed to detect drug sensitivity. The IC50 values for MMC, VCR, OXA and sorafenib in HepG2/IR cells were significantly higher than those in HepG2 cells at 48 and 72 h (Fig. 2A). The positive rate of EdU was significantly higher than that in the NC group (Fig. 2B). Moreover, flow cytometry with the Annexin V/PI double staining assay (Fig. 2C) revealed that HepG2/IR cells exhibited a significantly decreased cell apoptosis rate. Animal experimental results showed that the tumour volume of the HepG2/IR cell group was larger than that of the control group. In particular, mice in the HepG2 cell group treated with OXA had the minimum tumour volume (Fig. 2D), suggesting that IR could significantly inhibit OXA sensitivity in hepatoma cells.

### Overexpressed mir-5195-3p inhibited proliferation, migration, invasion, EMT and chemoresistance in HepG2/IR cells

**Fig. 3:**
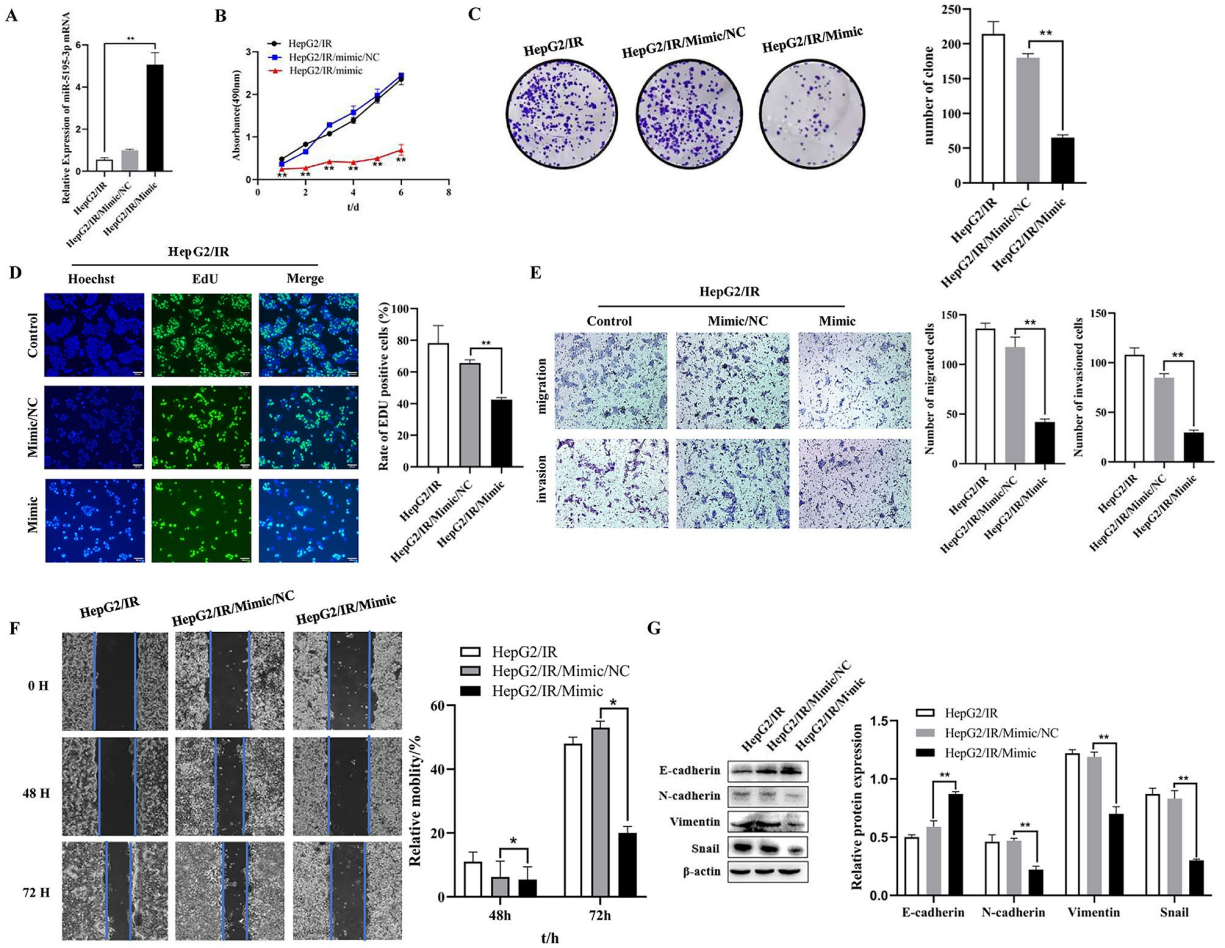
Overexpressed miR-5195-3p inhibited proliferation, migration, invasion, and EMT in HepG2/IR cells. (**A**). Relative miR-5195-3p expression levels in HepG2/IR cells were detected using qRT‒PCR after transfection with miR-5195-3p mimic or its control (NC mimic). MTT assays (**B**), colony formation assays (**C**), and EdU assays (**D**) were employed to detect the proliferation of HepG2/IR cells after transfection with the NC mimic or the miR-5195-3p mimic. Representative images of migration and invasion (**E**) and scratch wound healing assays (**F**) of HepG2/IR cells transfected with miR-5195-3p mimic or NC were determined. G. The protein expression of E-cadherin, N-cadherin, Vimentin and Snail in HepG2/IR cells transfected with miR-5195-3p mimic was determined by Western blots; the membranes were cut prior to hybridization with antibodies. The experiments were independently repeated three times. (* P < 0.05, ** P < 0.01)

As shown in Fig. 3A, the expression of miR-5195-3p was significantly increased after transfection with the miR-5195-3p mimic in HepG2/IR cells compared with that of the NC mimic group. The results of the MTT assay indicated that the overexpression of miR-5195-3p inhibited HepG2/IR cell proliferation (Fig. 3B). Moreover, a colony formation assay was performed. Decreased proliferation was found in the miR-5195-3p-overexpressing HepG2/IR cells (70.67 ± 2.52) compared with the NC controls (211.33 ± 4.04) (Fig. 3C). A reduced EdU-positive rate was found in the miR-5195-3p-overexpressing HepG2/IR cells (42.55 ± 1.23%) compared with the control cells (65.66 ± 2%) (Fig. 3D). To investigate the effects of miR-5195-3p on cell migration, invasion and EMT, we assessed HepG2/IR cells by wound-healing assays, transwell assays and Western blots. The transwell assay with or without Matrigel demonstrated that the miR-5195-3p mimic group showed significantly suppressed migration and invasion of HepG2/IR cells compared with the control groups (Fig. 3E). The 48 and 72 h wound-healing assays revealed that the migratory capacity was decreased significantly with the miR-5195-3p mimic compared with that of the control cells (Fig. 3F). As shown in Fig. 3G, miR-5195-3p overexpression significantly inhibited the protein expression of N-cadherin, Vimentin and Snail and promoted the protein expression of E-cadherin in HepG2/IR cells compared with the mimic NC cells. These results suggested that upregulation of miR-5195-3p interfered with proliferation, migration, invasion and EMT in HepG2/IR cells.

**Fig. 4:**
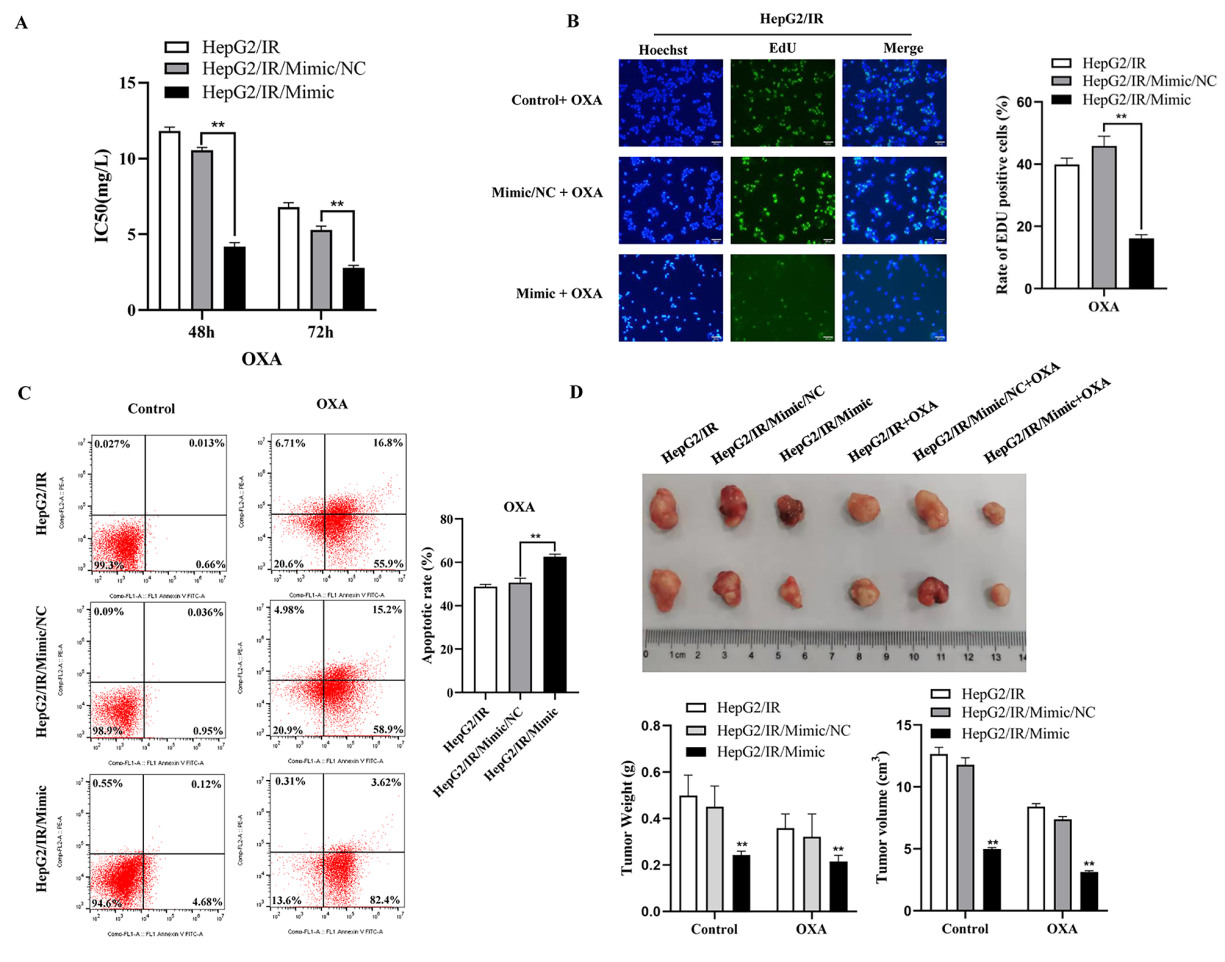
Overexpressed miR-5195-3p inhibited chemoresistance in HepG2/IR cells. The IC50 value (**A**) and EdU-positive rate (**B**) to OXA in HepG2/IR cells transfected with mimic or miR-NC. (**C**). Representative images of apoptosis. (**D**). The tumours dissected from all groups were photographed. The experiments were independently repeated three times. (* P < 0.05, ** P < 0.01)

To investigate and illuminate the relationship between miR-5195-3p and chemoresistance in HepG2/IR cells, we performed cell viability assays, EdU assays, apoptotic analyses, and in vivo animal experiments to assess the sensitivity of drug-resistant HepG2/IR cells to OXA after miR-5195-3p was upregulated. The IC50 values for OXA in HepG2/IR cells were significantly lower in the mimic group than in the miR-NC group (Fig. 4A). The positive rate of EdU was also remarkably lower than that in the NC group (Fig. 4B). Moreover, flow cytometry with the Annexin V/PI double staining assay (Fig. 4C) revealed that miR-5195-3p mimic transfection significantly increased cell apoptosis in HepG2/IR cells in comparison with the results of the miR-NC transfected group. Animal experimental results showed that the tumour volume of the miR-5195-3p mimic group was smaller than that of the mock or control group. In particular, mice in the miR-5195-3p mimic group treated with OXA had the minimum tumour volume (Fig. 4D). These findings suggested that miR-5195-3p could significantly elevate the OXA sensitivity of HepG2/IR cells.

### Inhibited mir-5195-3p promoted proliferation, migration, invasion, EMT and chemoresistance in HepG2 cells

**Fig. 5:**
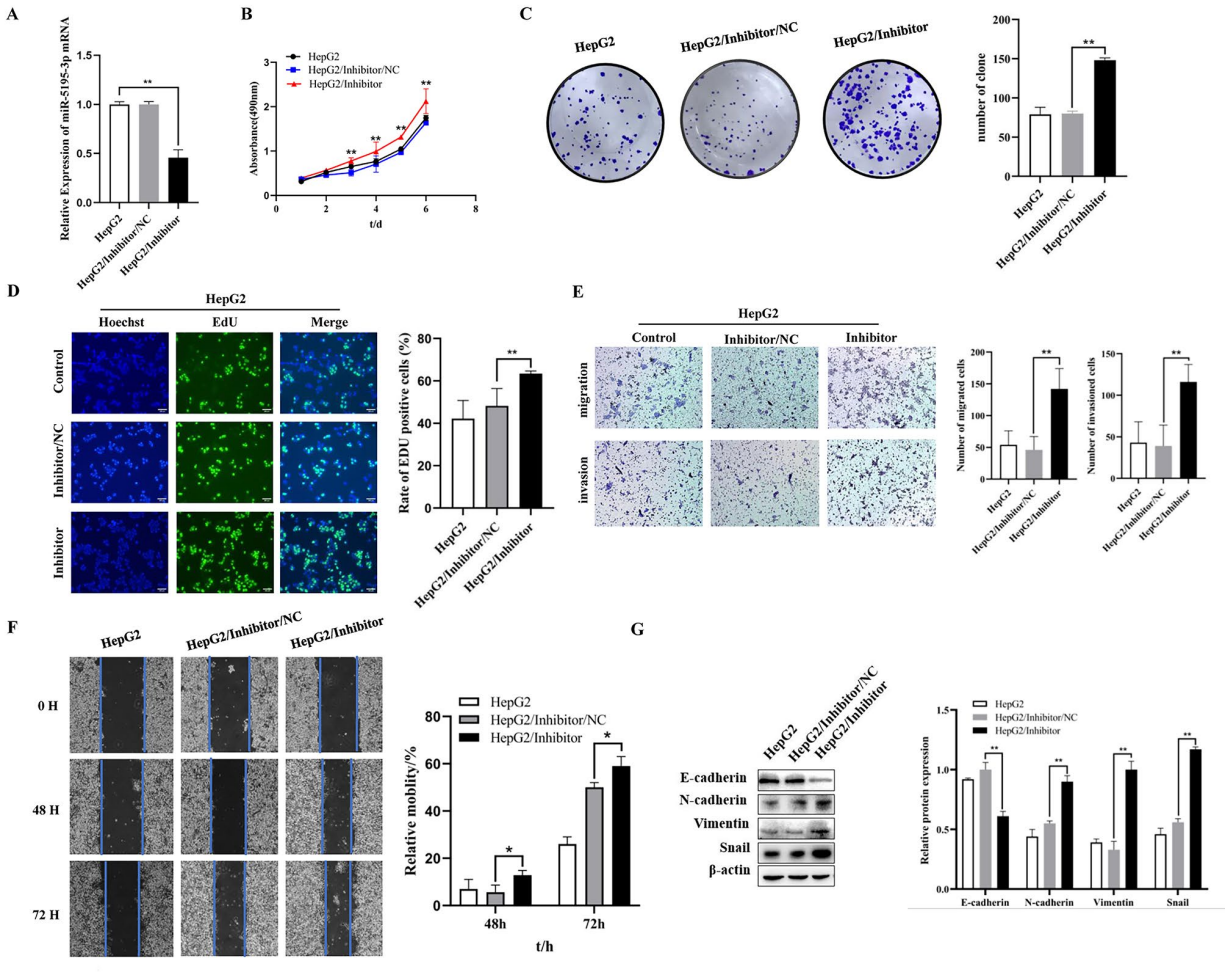
Inhibited miR-5195-3p promoted proliferation, migration, invasion, and EMT in HepG2 cells. (**A**). Relative miR-5195-3p expression levels in HepG2 cells were detected using qRT‒PCR after transfection with miR-5195-3p inhibitor or its control (NC inhibitor). MTT assays (**B**), colony formation assays (**C**), and EdU assays (**D**) were employed to detect the proliferation of HepG2 cells after transfection with the NC inhibitor or the miR-5195-3p inhibitor. Representative images of migration and invasion (**E**) and scratch wound healing assays (**F**) of HepG2 cells transfected with miR-5195-3p inhibitor or NC were determined. (**G**). The protein expression of E-cadherin, N-cadherin, Vimentin and Snail in HepG2 cells transfected with miR-5195-3p inhibitor was determined by Western blotting; the membranes were cut prior to hybridization with antibodies. The experiments were independently repeated three times. (* P < 0.05, ** P < 0.01)

As shown in Fig. 5A, the expression of miR-5195-3p was significantly decreased after transfection with the miR-5195-3p inhibitor in HepG2 cells compared with that of the NC inhibitor group. (Fig. 5A) An MTT assay was employed to detect the proliferation of HepG2 cells transfected with miR-5195-3p inhibitor or NC inhibitor. The results indicated that downregulating the expression of miR-5195-3p elevated proliferation of HepG2 cells (Fig. 5B). Moreover, colony formation assays showed increased proliferation of miR-5195-3p-downregulated HepG2 cells (145.67 ± 13.05) compared with the NC controls (76.33 ± 4.51) (Fig. 5C). In addition, an enhanced EdU-positive rate was found in the miR-5195-3p-downregulated HepG2 cells compared with the controls (Fig. 5D). To investigate the effects of miR-5195-3p on cell migration, invasion and EMT, we assessed cells by wound-healing assays, transwell assays and Western blots, respectively. Transwell assays with or without Matrigel demonstrated that the miR-5195-3p inhibitor group exhibited significantly increased migration and invasion compared with the control group (Fig. 5E). The 48 and 72 h wound-healing assays also revealed that the migratory capacity was elevated significantly (Fig. 5F). As shown in Fig. 5G, downregulation of miR-5195-3p significantly increased the protein expression of N-cadherin, Vimentin and Snail and suppressed the expression of E-cadherin in HepG2/IR cells compared with those of the inhibitor NC group. These results suggested that downregulated miR-5195-3p induced enhanced proliferation, migration, invasion and EMT in HepG2 cells.

**Fig. 6:**
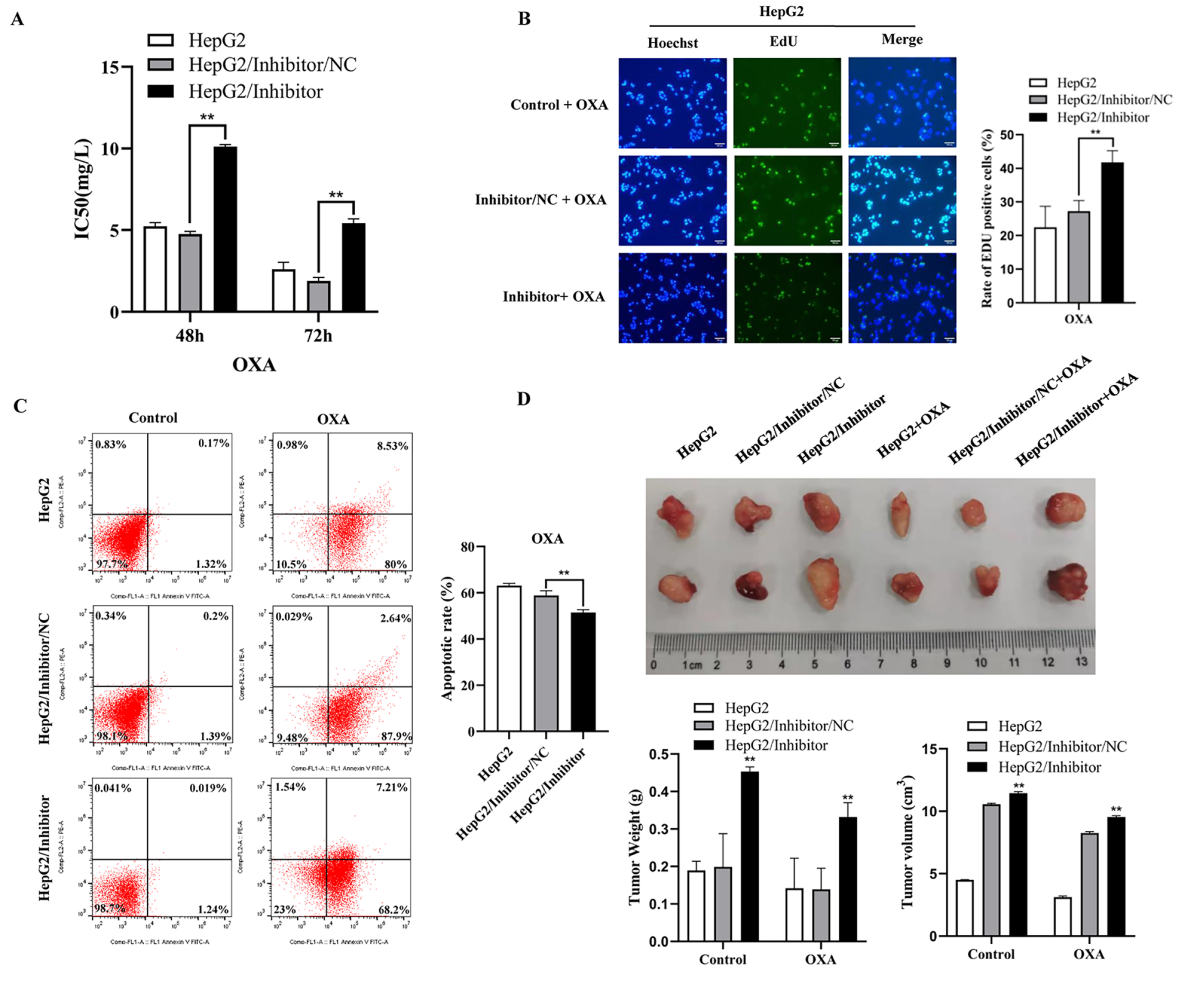
Inhibition of miR-5195-3p promoted chemoresistance in HepG2 cells. The IC50 value (**A**) and EdU-positive rate (**B**) for OXA in HepG2 cells transfected with inhibitor or miR-NC. (**C**). Representative images of apoptosis. (**D**). The tumours dissected from all groups were photographed. The experiments were independently repeated three times. (* P < 0.05, ** P < 0.01)

To investigate and illuminate the relationship between miR-5195-3p and chemoresistance in HepG2 cells, we performed a cell viability assay, an EdU assay, apoptosis analysis, and in vivo animal experiment to detect sensitivity to OXA in HepG2 cells after downregulating the expression of miR-5195-3p. The IC50 value of OXA in HepG2 cells was clearly higher in the inhibitor group than in the miR-NC group (Fig. 6A). The positive rate of EdU was also significantly higher than that in the miR-NC group (Fig. 6B). Moreover, flow cytometry with the Annexin V/PI double staining assay revealed that inhibition of miR-5195-3p could remarkably decrease cell apoptosis in HepG2 cells in comparison with the results for miR-NC transfection (Fig. 6C). Animal experimental results showed that the tumour volume of the miR-5195-3p inhibitor group was larger than that of the mock or control group (Fig. 6D). These findings suggested that miR-5195-3p could significantly decrease the OXA sensitivity of HepG2 cells.

### Mir-5195-3p regulated the expression of SOX9 and TPM4 by directly targeting their 3′-UTRs

**Fig. 7:**
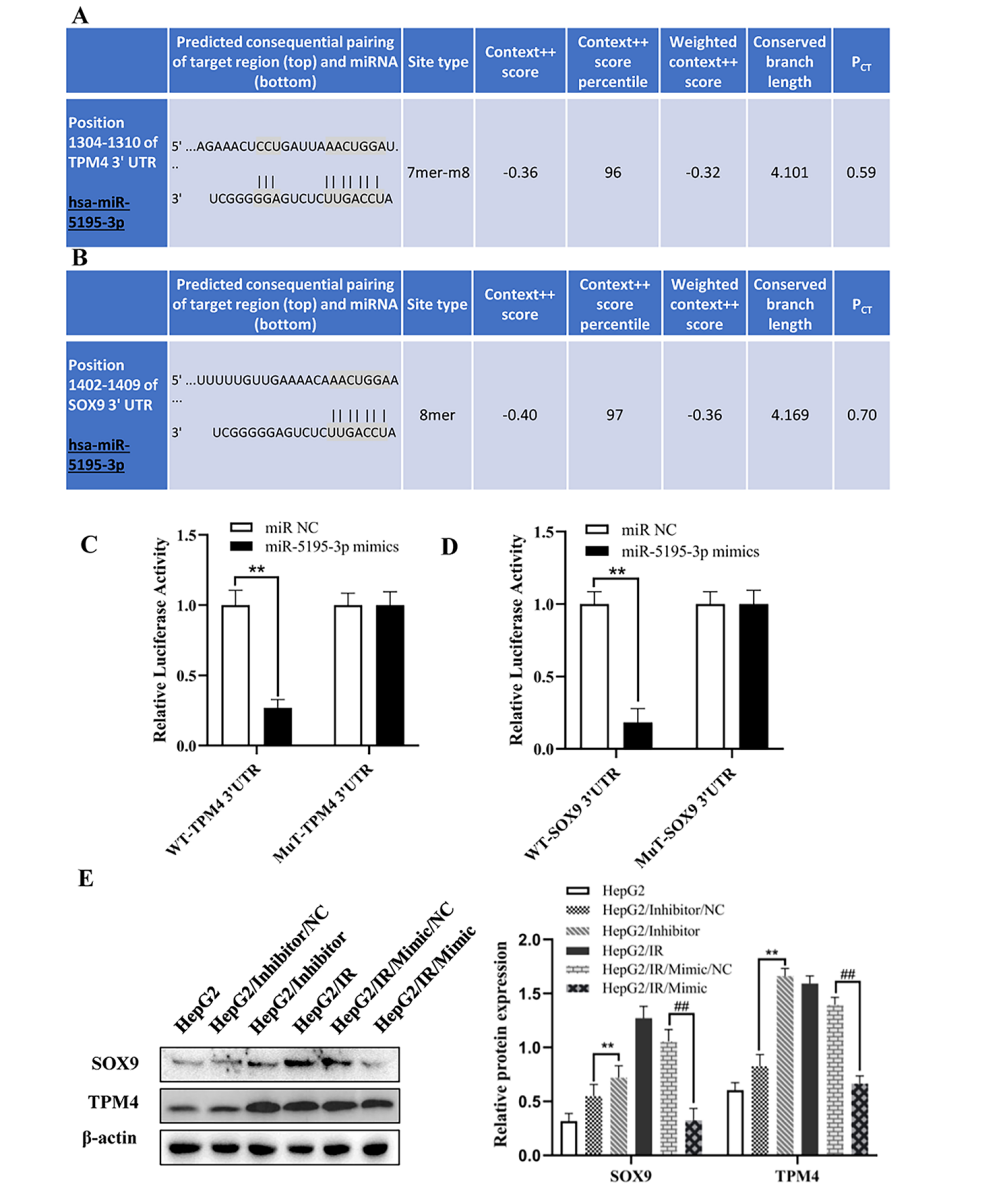
miR-5195-3p directly targeted the 3′-UTR of SOX9 and TPM4. The predicted targeting sites of SOX9 and TPM4 (**A** and **B**). A dual luciferase reporter assay was performed to verify the direct target of miR-5195-3p (**C** and **D**). (**E**). Western blotting was performed to detect the protein expression levels of SOX9 and TPM4 in HepG2 cells transfected with NC inhibitor or miR-5195-3p inhibitor, and HepG2/IR cells were transfected with the NC mimic or miR-5195-3p mimic; the membranes were cut prior to hybridization with antibodies. The experiments were independently repeated three times. (* P < 0.05, ** P < 0.01)

To identify the potential targets of miR-5195-3p, we used bioinformatics strategies. The SOX9 and TPM4 3′-UTRs that were predicted by TargetScan had target sites for miR-5195-3p (Fig. 7A, B). The luciferase reporter assay results exhibited significantly decreased luciferase activity in the wild-SOX9-3′UTR (WT) and WT-TPM4-3′UTR groups compared with the control groups, but the mutant reporters (MT) were not repressed by miR-5195-3p (Fig. 7C, D). In addition, Fig. 7E not only shows that SOX9 and TPM4 were upregulated in HepG2/IR cells compared with HepG2 cells but also reveals that overexpression of miR-5195-3p suppressed the expression of SOX9 and TPM4 in HepG2/IR cells, which suggested that miR-5195-3p targeted the 3′UTR of SOX9 and TPM4 directly and regulated the expression of SOX9 and TPM4.

## Discussion

Liver cancer is one of the most common types of cancer. During the past decades, surgery, chemotherapy, and immunotherapy have shown major advances in treating hepatoma. However, the majority of HCC patients have already developed distant metastases and do not respond to the treatments mentioned above, resulting in a poor prognosis and long-term survival rate following surgical resection [21]. To develop more effective diagnosis and treatment strategies, researchers must gain a deeper understanding of the mechanisms of malignancy in liver cancer.

Chronic IR conditions occur when the sensitivity to insulin decreases, as well as the ability to absorb and utilize glucose within organisms or cells. It has been proven that IR is an independent risk factor that accelerates the progression of liver cancer [22]. The mechanism leading to the enhancement of malignant biological behaviour of IR HCC is still unclear. In this study, HepG2 cells were induced with a high concentration (0.2 µmol/L) of insulin for a long time (72 h) to produce stable IR. After a series of in vitro functional experiments, such as MTT analysis, cell scratch tests and flow cytometry, as well as in vivo nude mouse xenograft experiments, it was found that the proliferation, migration, invasion, EMT ability and chemoresistance of HepG2/IR cells were significantly enhanced, indicating that IR could promote stronger malignant biological behaviour in HCC.

Studies have shown that miRNAs can participate in various biological processes, such as proliferation, invasion, EMT and drug tolerance, in numerous cancers through negative regulation of gene expression [23–25]. It was confirmed that miR-5195-3p inhibited the proliferation and invasion of bladder cancer cells by targeting the oncogene KLF5.21 and inhibited the activity of HCT116 cells by inhibiting the expression of TGFβR1, TGFβR2, SMAD3 and SMAD4 [26]. Numerous studies have shown that miR-5195-3p plays a significant role in several cancers [27–29]. However, the regulatory mechanism of miR-5195-3p in IR liver cancer cells remains unclear. Oxaliplatin (OXA), the anticancer drug we have chosen, is an extensively used anticancer medicine worldwide, and the clinical activity of several schemes containing it in advanced HCC has been demonstrated in recent studies [30, 31]. Consistent with other studies, we proved that impaired expression of miR-5195-3p in IR hepatoma cells was involved in malignant biological processes such as proliferation, migration, EMT and drug resistance, and the results were contrary. To clarify the function of miR-5195-3p in vivo, we performed tumour formation experiments in nude mice in HepG2/IR cells transfected with miR-5195-3p mimics and HepG2 cells transfected with miR-5195-3p inhibitors. The same results were observed in vitro.

We also verified that SOX9 and TPM4 were the targets of miR-5195-3p. Thus, we speculated that miR-5195-3p regulates the malignant behaviour of IR hepatoma cells. SOX9 is expressed in a variety of cancers, including pancreatic cancer, breast cancer, and prostate cancer [18]. Studies have shown that SOX9 can interact with a variety of downstream proteins and exhibit stimulatory or inhibitory activity in different types of tumour cells [32, 33]. It was also reported that SOX9 could activate the Wnt/β-catenin pathway to drive the growth and metastasis of gastric cancer [34]. In addition, studies have shown that SOX9 can be negatively regulated by microRNAs and regulate the development of cancer. For example, miR-216b inhibited the proliferation and invasion of non-small cell lung cancer (NSCLC) cells by directly targeting SOX9, and miR-145 reduced the adhesion and invasion of glioblastoma cells by inhibiting the carcinogenic proteins of SOX9 and ADD3 [35, 36]. As an actin-binding protein, TPM4 belongs to the protomyosin family, which can enhance the migration of tumour cells by changing the actin cytoskeleton [19]. TPM4 was found to be abnormally expressed in a variety of cancers, and it was considered to be a potential biomarker for liver cancer, ovarian cancer, colon cancer, etc. [37–39]. Studies have shown that enhanced TPM4 could promote the migration of certain types of cancer cells without participating in cell proliferation and EMT progression [40]. Our previous study also found that TPM4 was highly expressed in HCC tissues and HCC cells with high invasiveness. In addition, TPM4 positivity was closely related to clinical pT grade, pathological grade and clinical stage [41].

## Conclusions

In summary, we found that downregulated miR-5195-3p could participate in the proliferation, invasion, EMT and chemoresistance of HepG2/IR cells by upregulating the expression of the target genes SOX9 and TPM4. Our findings provide new insights into the molecular function of miR-5195-3p and a potential therapeutic target in liver cancer. Finally, this study is limited, as we restricted our work to HepG2 liver cancer cells. Further studies should be performed on liver cancer cells with miR-5195-3p.

## Electronic supplementary material

Below is the link to the electronic supplementary material.

Supplementary Material 1

## Data Availability

The datasets generated and/or analysed during the current study are not publicly available due [Internal policy of The Second Hospital of Lanzhou University] but are available from the corresponding author on reasonable request.
