# Supplementary material for: MicroRNA-5195-3p mediated malignant biological behaviour of insulin-resistant liver cancer cells via SOX9 and TPM4
Source: BMC Cancer. 2023 Jun 16;23:557. doi: 10.1186/s12885-023-11068-x (PMC10273698; doi:10.1186/s12885-023-11068-x)
Supplement: Supplementary file 1 — Supplementary Material 1 [file 12885_2023_11068_MOESM1_ESM.pdf]

Three gels

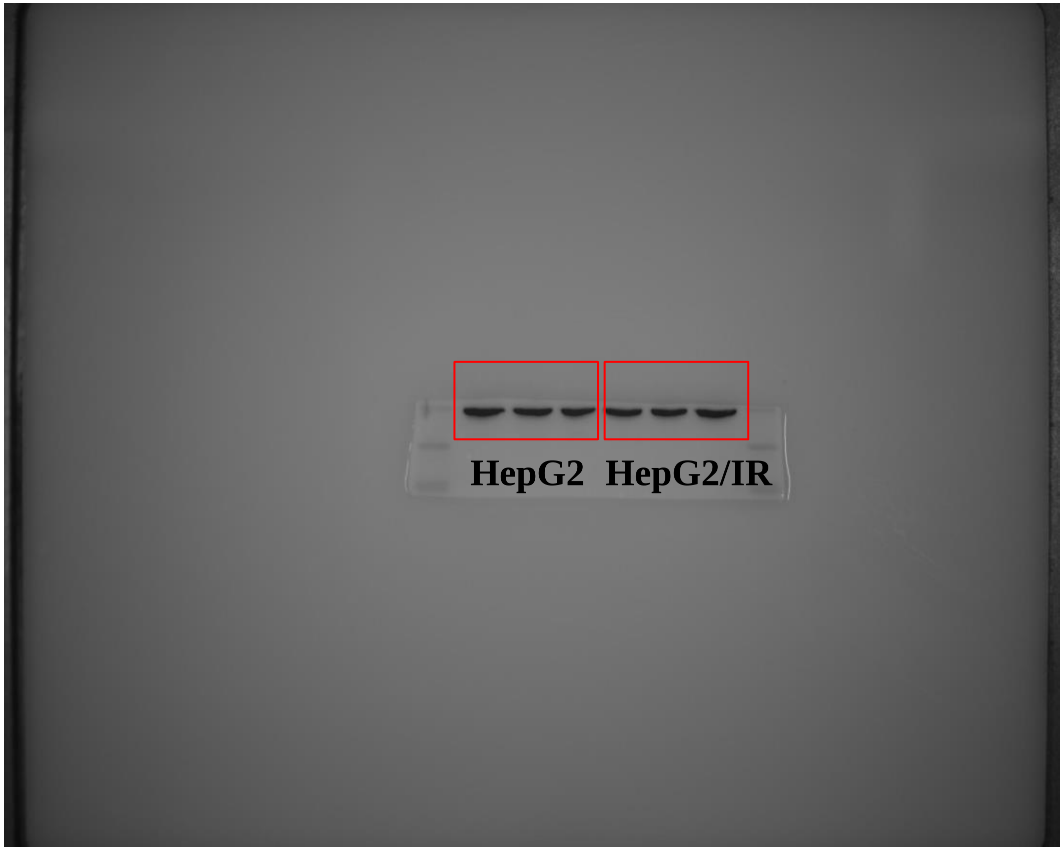

β-actin

Three gels

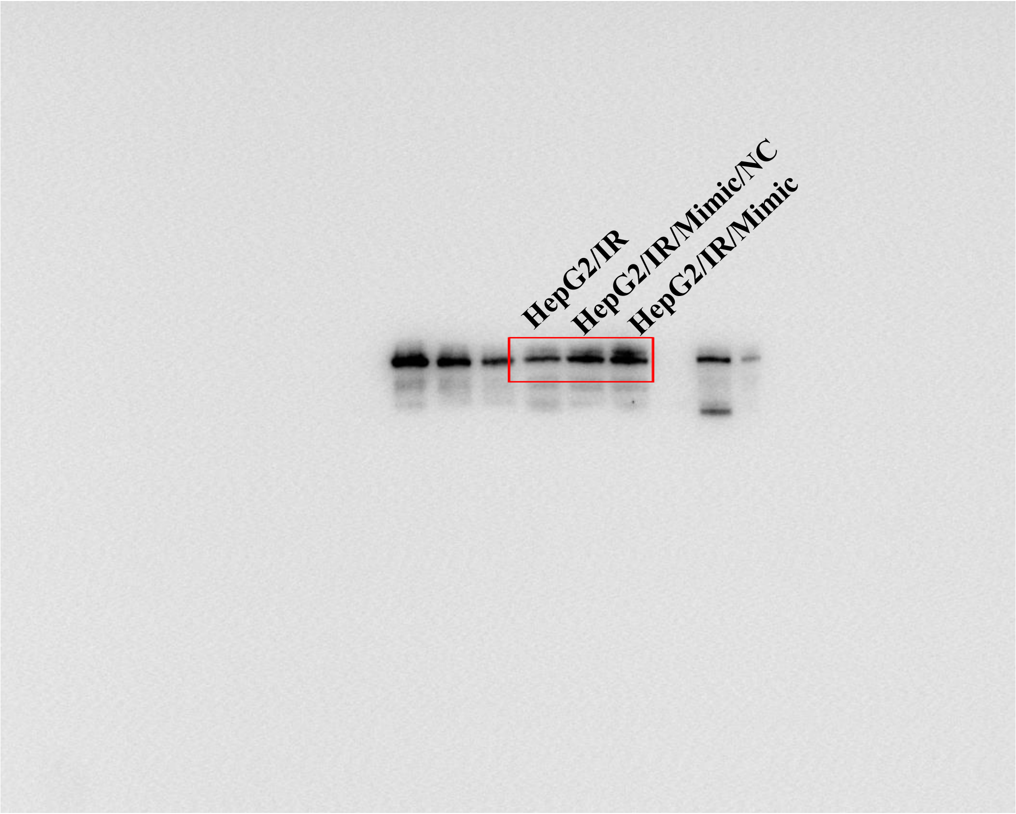

E-cadherin

Three gels

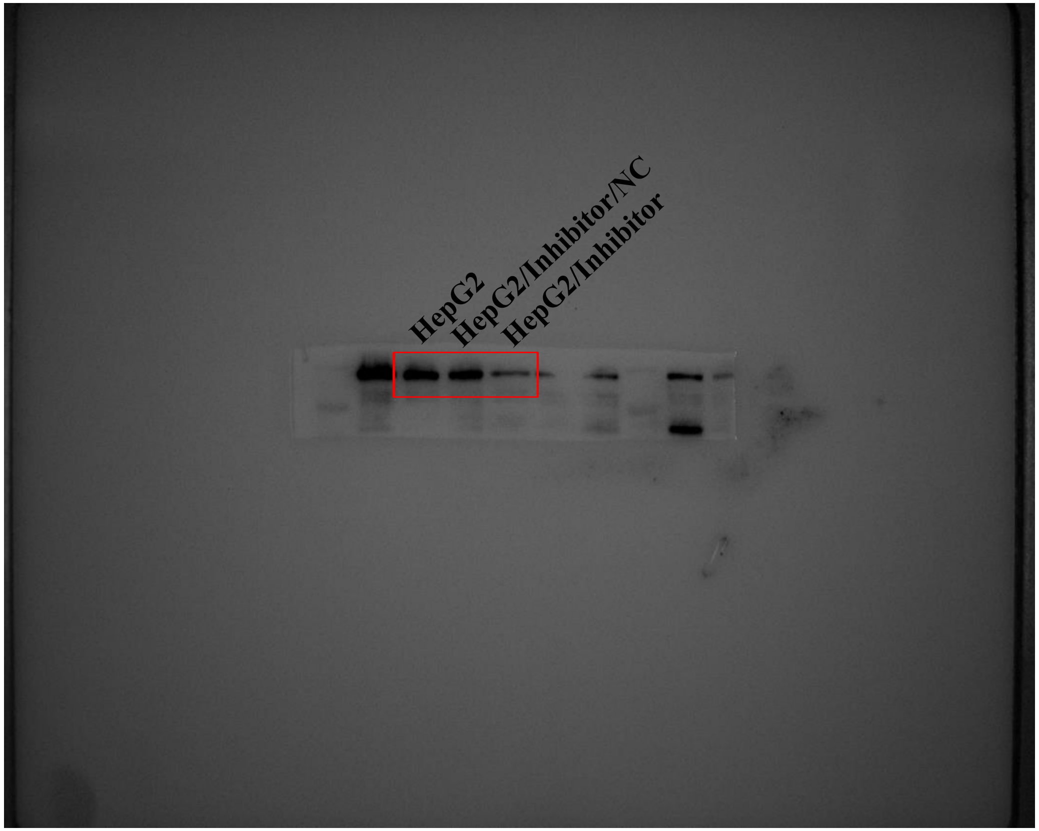

E-cadherin

Six gels

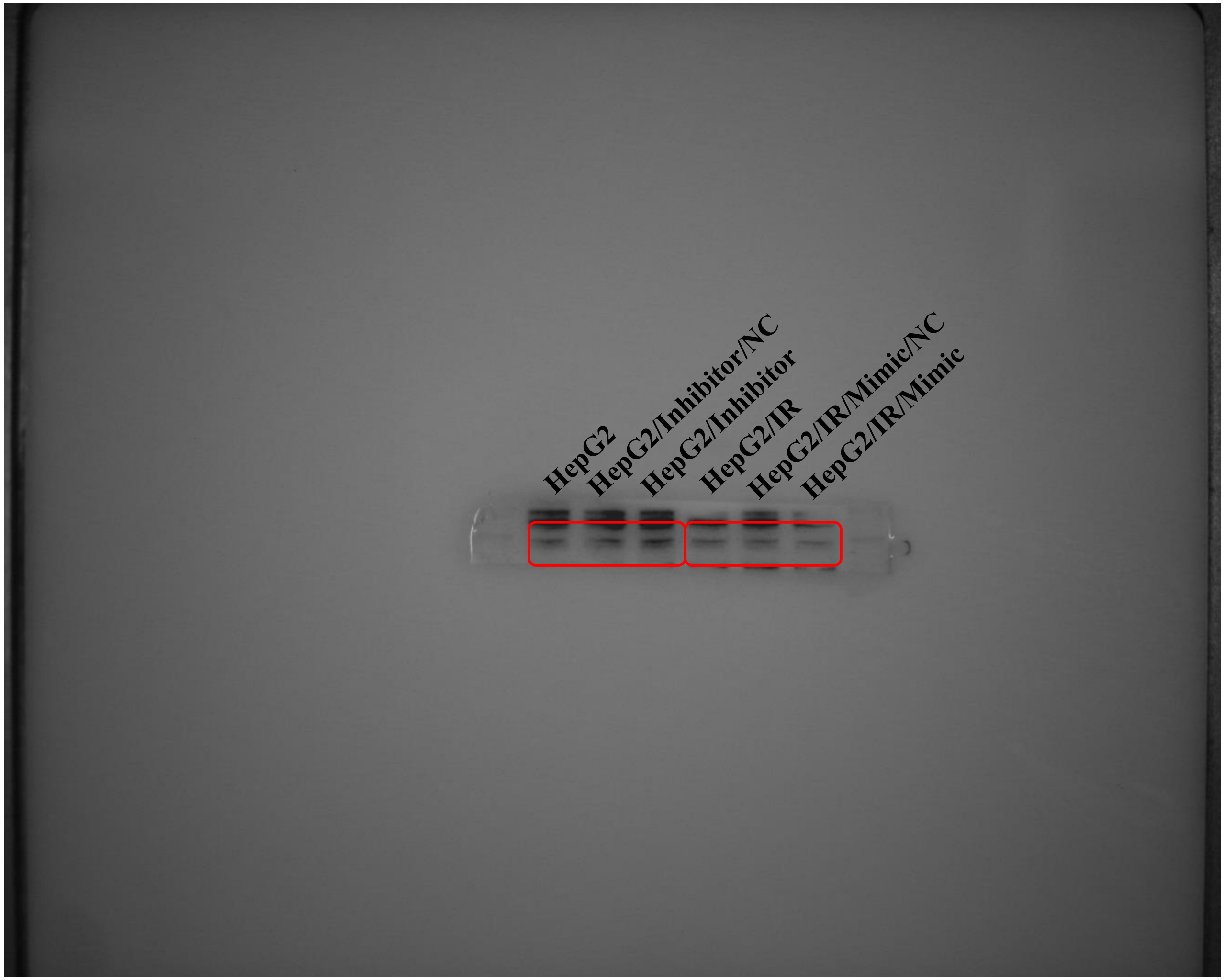

N-cadherin

Three gels

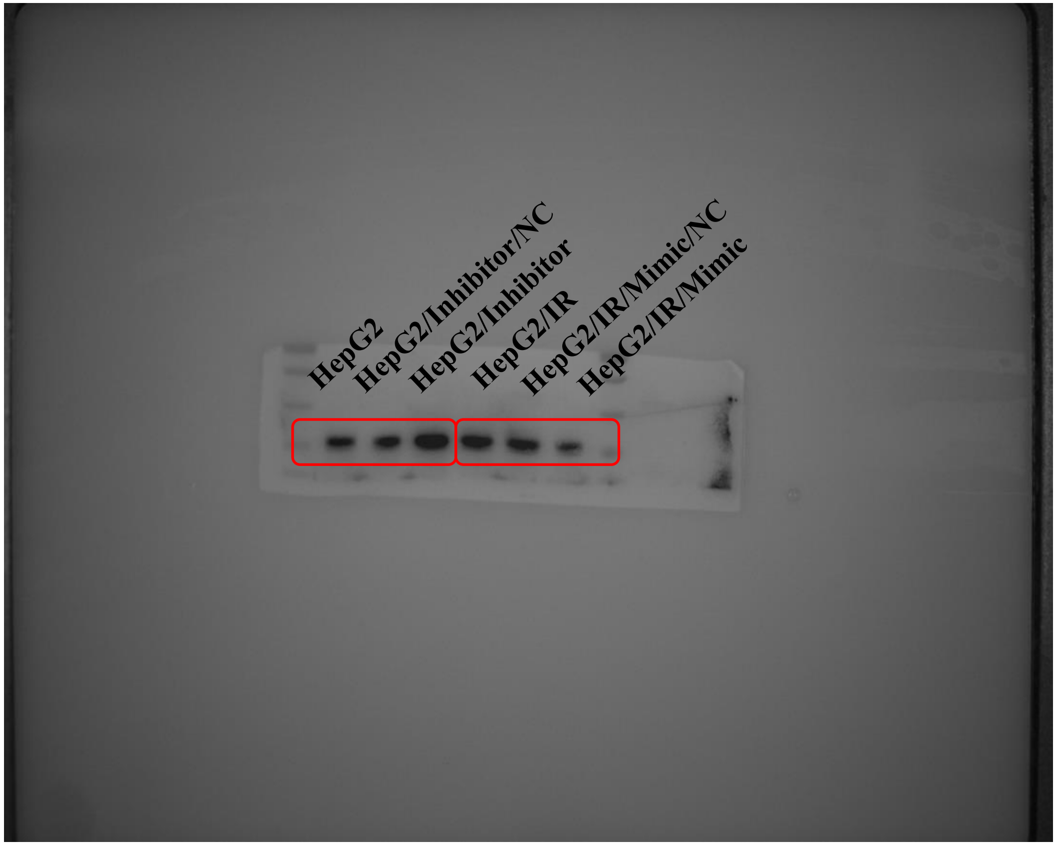

Snail

Three gels

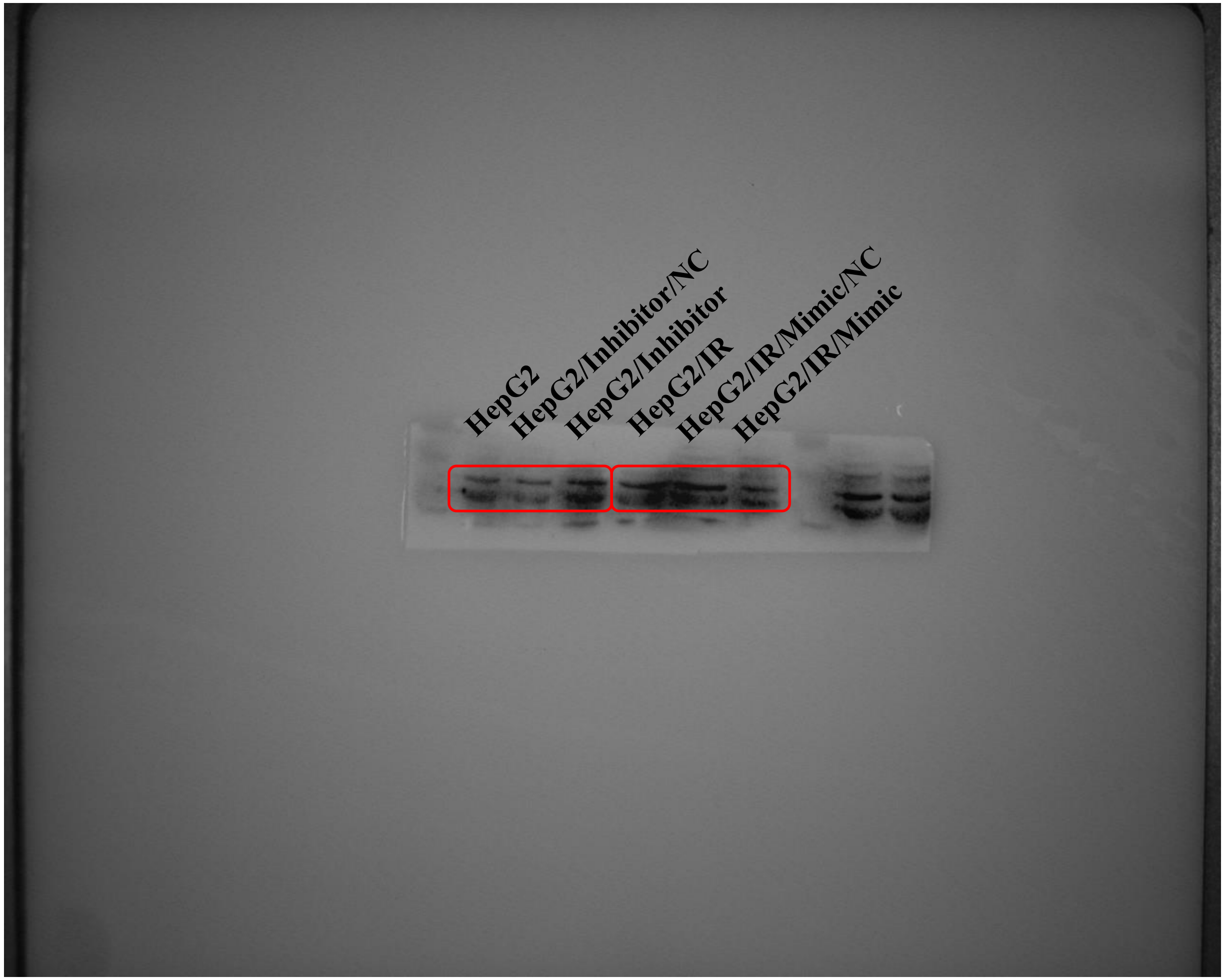

Vimentin

Three gels

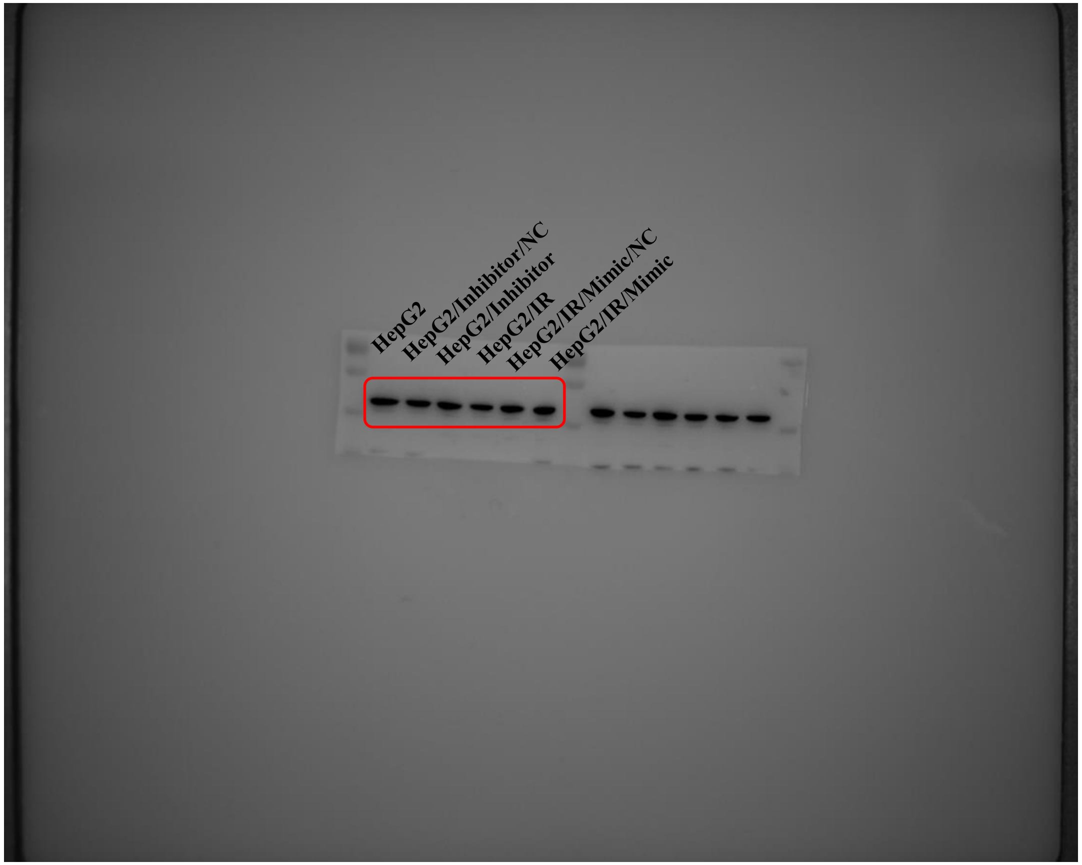

β-actin

Three gels

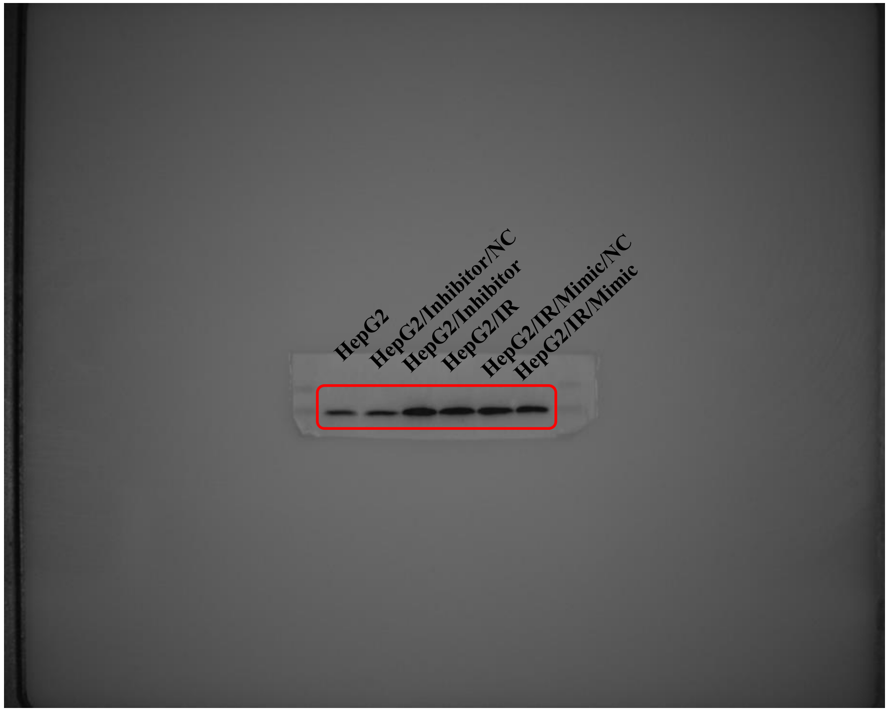

TPM4

Three gels

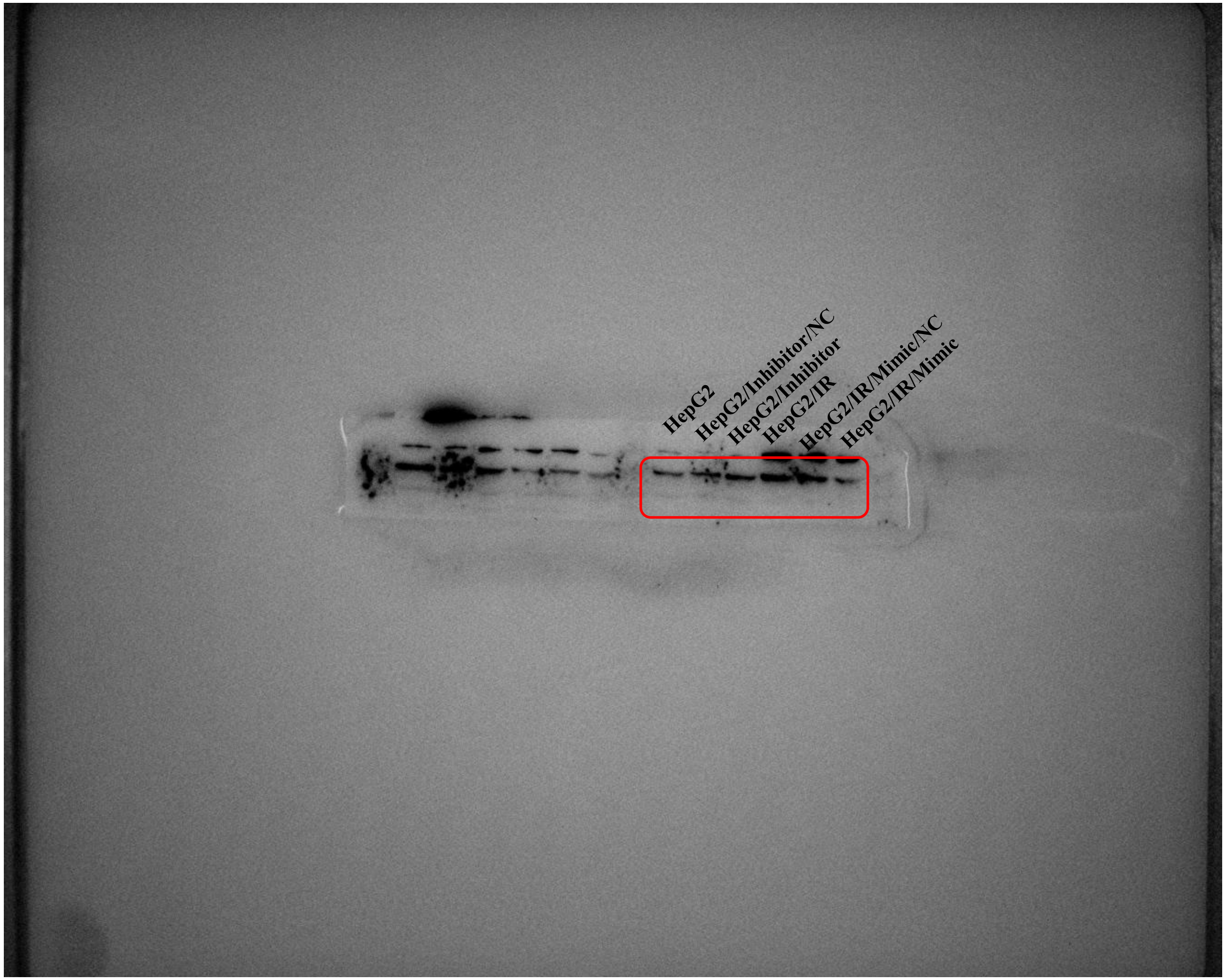

SOX9

Two gels

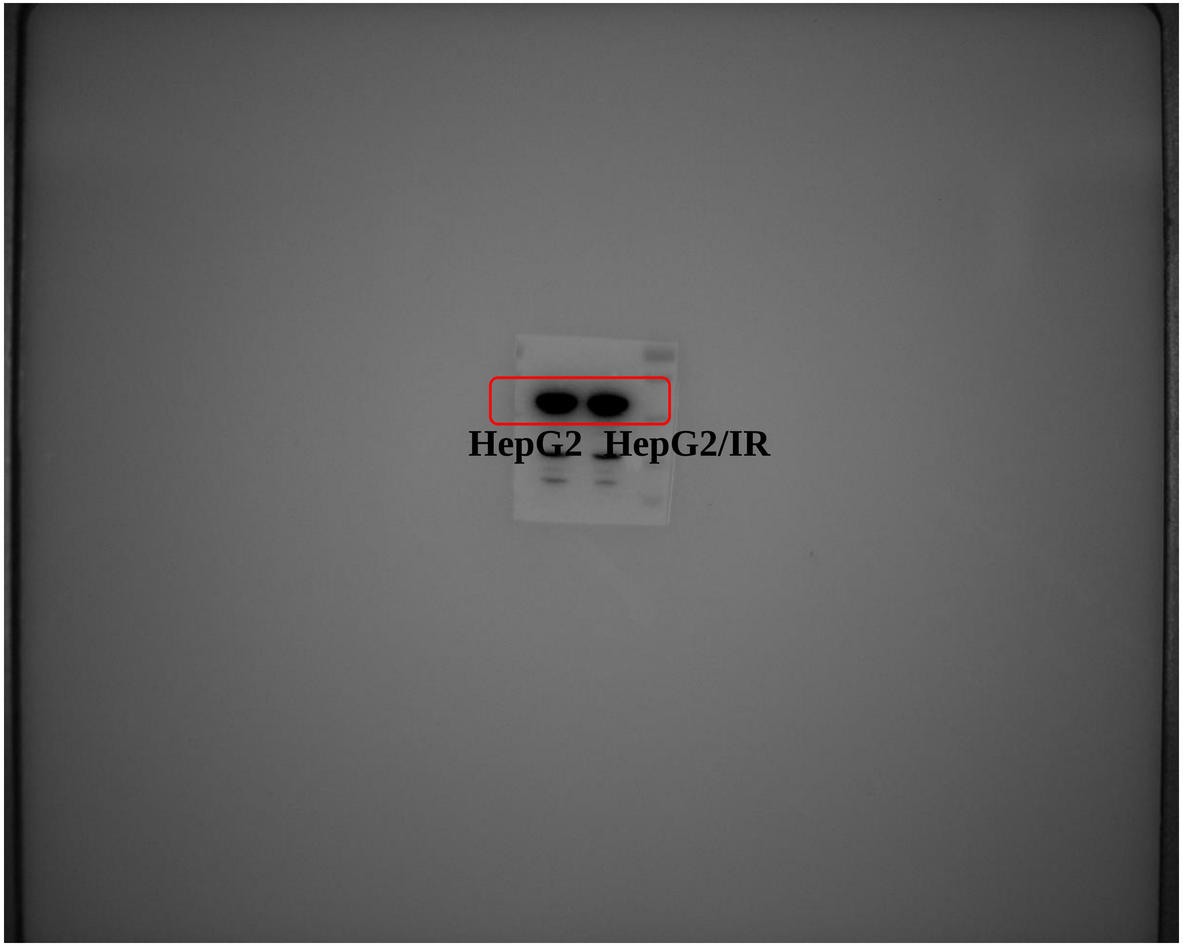

β-actin

Two gels

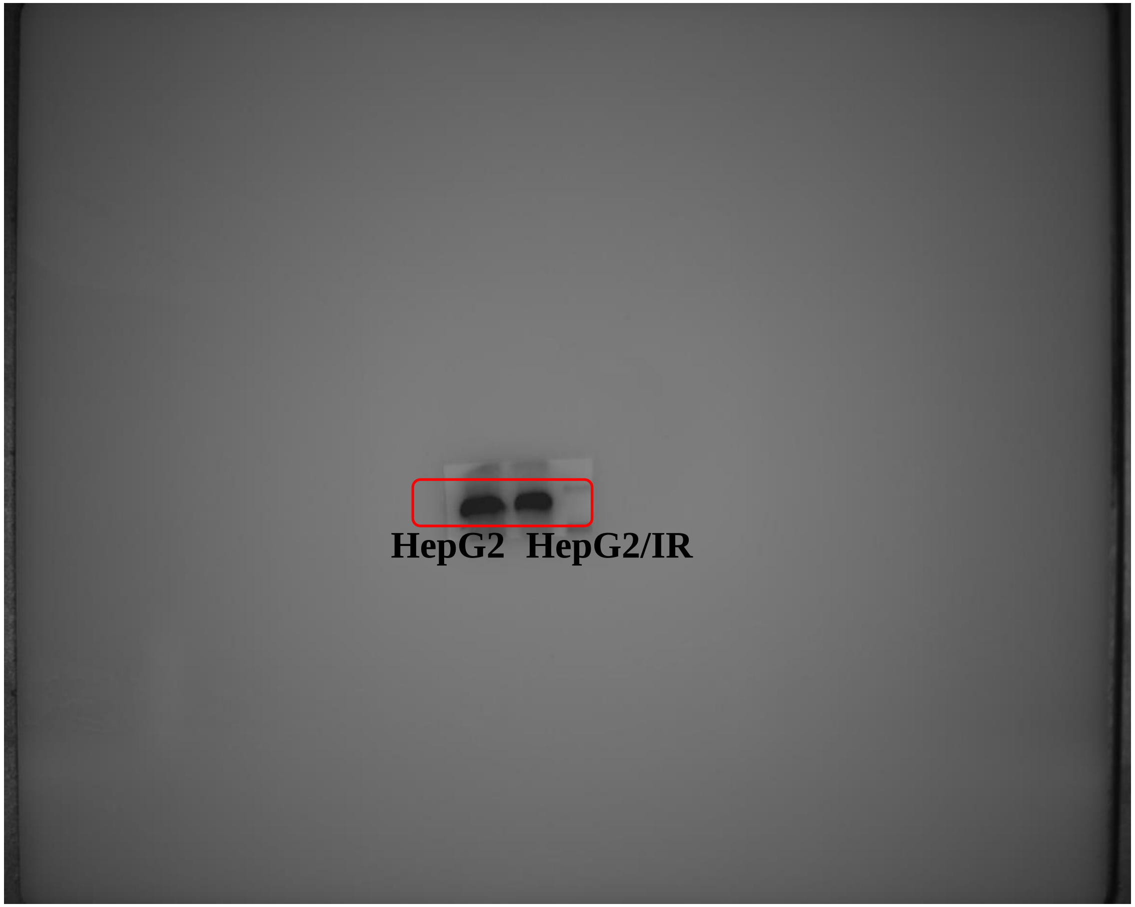

E-cadherin

Two gels

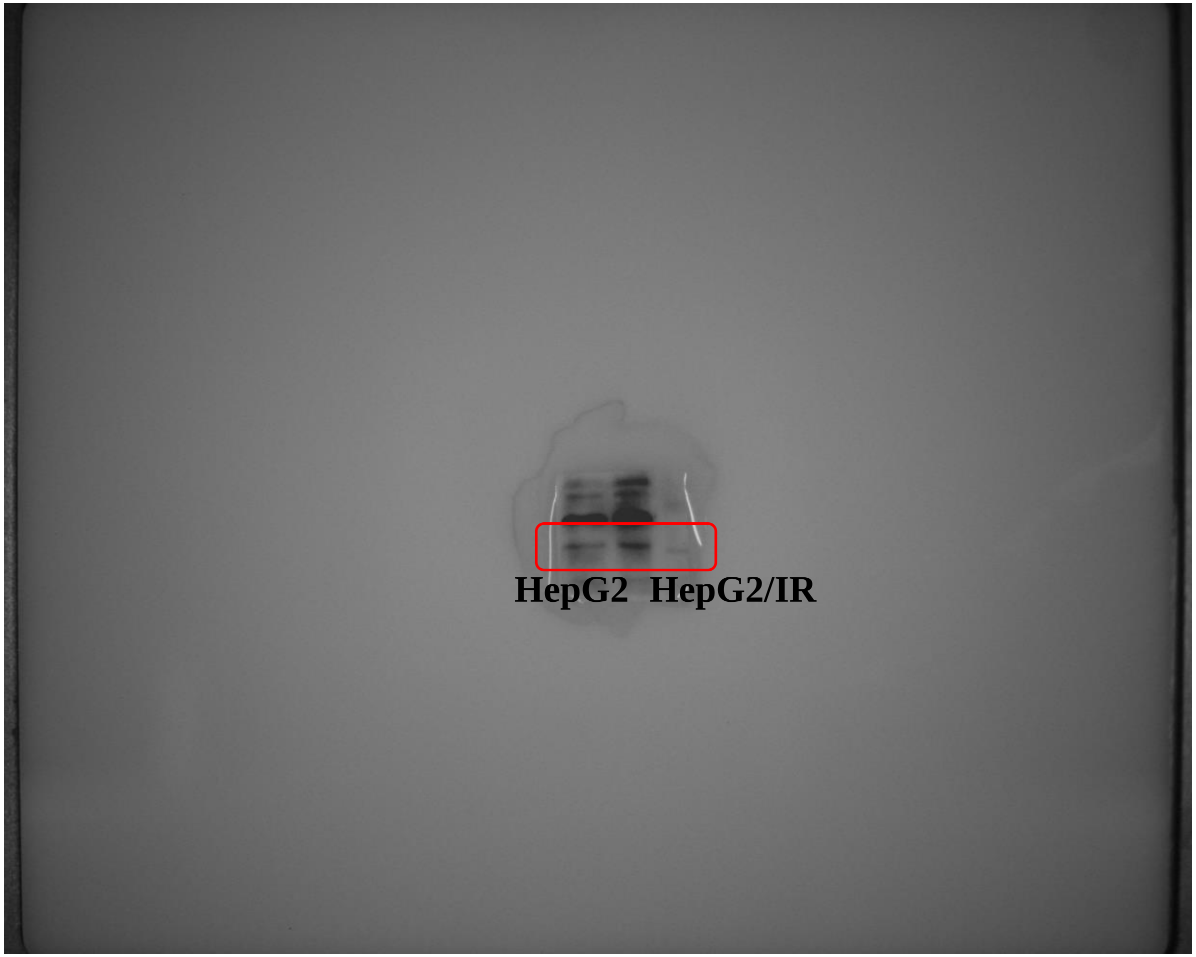

N-cadherin

Two gels

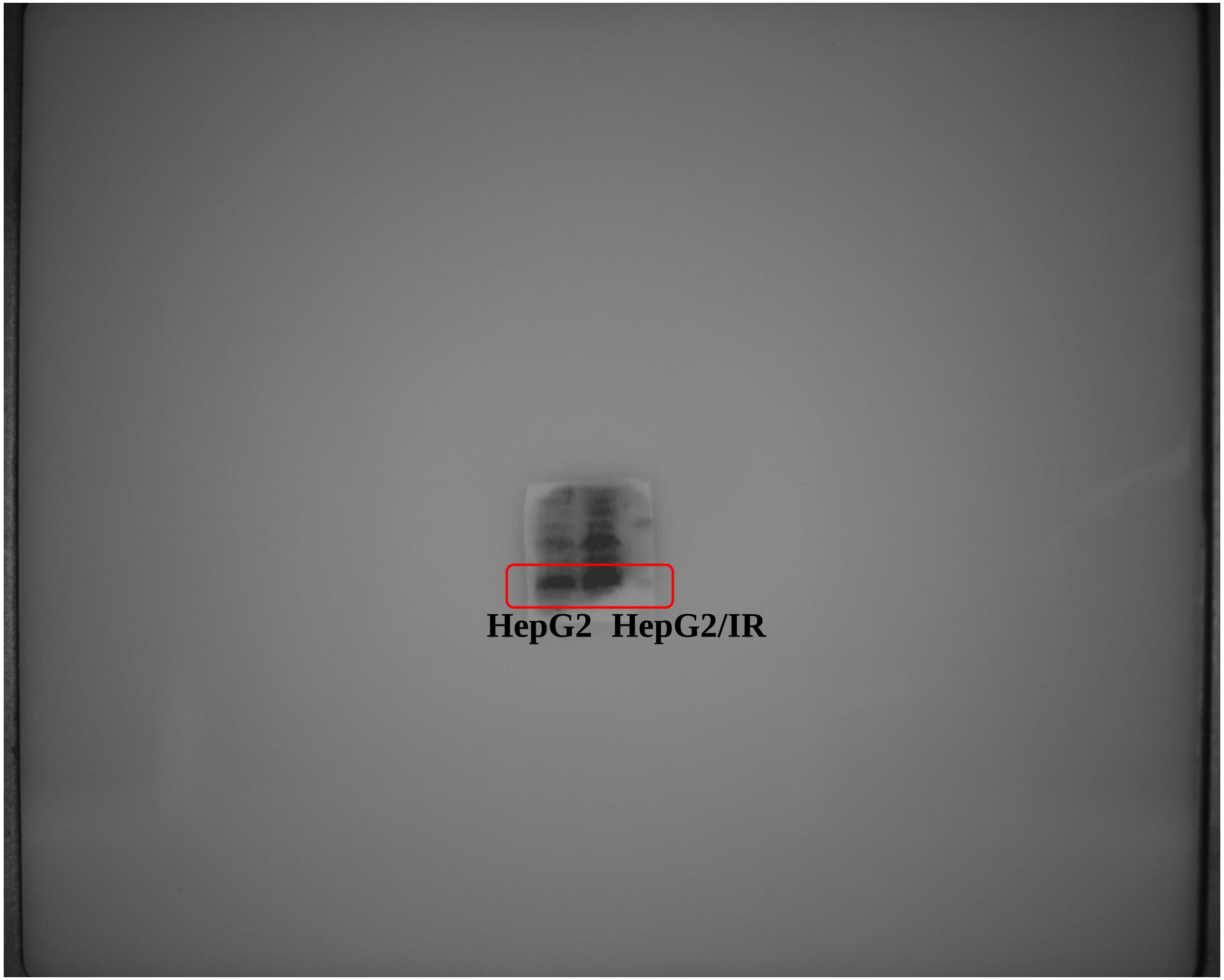

Vimentin

Two gels

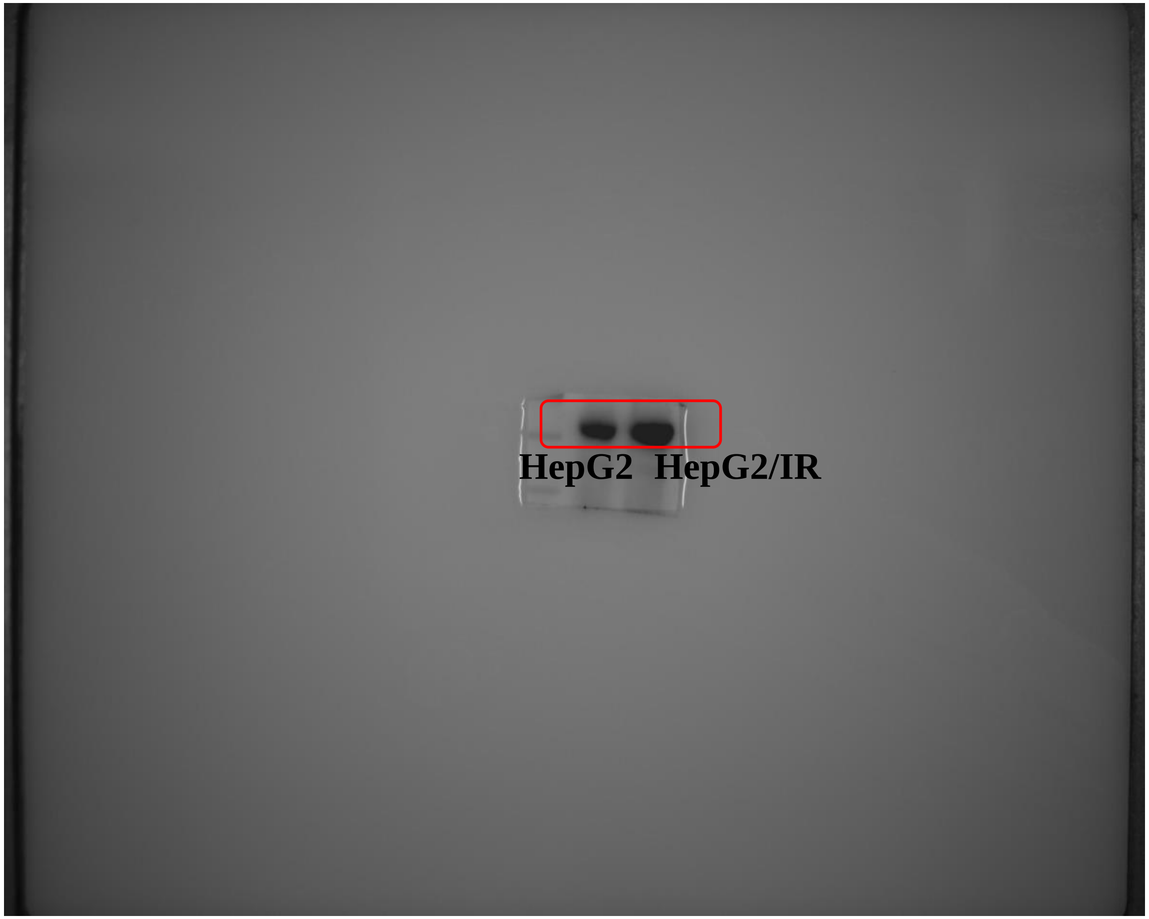

Snail
